# Genome-wide screening and characterization of long non-coding RNAs involved in flowering development of trifoliate orange (*Poncirus trifoliata* L. Raf.)

**DOI:** 10.1038/srep43226

**Published:** 2017-02-24

**Authors:** Chen-Yang Wang, Sheng-Rui Liu, Xiao-Yu Zhang, Yu-Jiao Ma, Chun-Gen Hu, Jin-Zhi Zhang

**Affiliations:** 1 Key Laboratory of Horticultural Plant Biology (Ministry of Education), College of Horticulture and Forestry Science, Huazhong Agricultural University, Wuhan 430070, China

## Abstract

Long non-coding RNAs (lncRNAs) have been demonstrated to play critical regulatory roles in post-transcriptional and transcriptional regulation in *Arabidopsis*. However, lncRNAs and their functional roles remain poorly characterized in woody plants, including citrus. To identify lncRNAs and investigate their role in citrus flowering, paired-end strand-specific RNA sequencing was performed for precocious trifoliate orange and its wild-type counterpart. A total of 6,584 potential lncRNAs were identified, 51.6% of which were from intergenic regions. Additionally, 555 lncRNAs were significantly up-regulated and 276 lncRNAs were down-regulated in precocious trifoliate orange, indicating that lncRNAs could be involved in the regulation of trifoliate orange flowering. Comparisons between lncRNAs and coding genes indicated that lncRNAs tend to have shorter transcripts and lower expression levels and that they display significant expression specificity. More importantly, 59 and 7 lncRNAs were identified as putative targets and target mimics of citrus miRNAs, respectively. In addition, the targets of Pt-miR156 and Pt-miR396 were confirmed using the regional amplification reverse-transcription polymerase chain reaction method. Furthermore, overexpression of Pt-miR156a1 and Pt-miR156a1 in *Arabidopsis* resulted in an extended juvenile phase, short siliques, and smaller leaves in transgenic plants compared with control plants. These findings provide important insight regarding citrus lncRNAs, thus enabling in-depth functional analyses.

Transcriptome sequencing in various organisms has revealed that extensive transcription derived from approximately 90% of the genome generates a large proportion of non-coding RNAs (ncRNAs)^1^. The ncRNAs are classified into two types: housekeeping ncRNAs, which consist of rRNAs, tRNAs, small nucleolar RNAs, and small nuclear RNAs, and regulatory ncRNAs, which include microRNAs (miRNAs), small interfering RNAs (siRNAs), and long non-coding RNAs (lncRNA)^2^,^3^. The lncRNAs, with lengths longer than 200 nucleotides, are devoid of open reading frames (ORFs) and are often polyadenylated^4^. The importance of lncRNAs has been immensely underestimated in early studies because of their low expression, low sequence conservation compared with mRNAs, and their designation as transcriptional noise^5^. Accumulating evidence indicates that lncRNAs play critical roles in various biological processes in animals and plants^4^,^5^. Recently, our understanding of the biological functions of lncRNAs has experienced a large step forward in mammals; however, studies investigating the functions of lncRNAs in plants are still in their infancy, especially those regarding their functions during reproduction^3^,^5^.

Like protein-coding genes, the majority of lncRNAs are transcribed by RNA polymerase II with a 5′ cap and a 3′ poly-A tail in animals^6^. However, lncRNAs can be transcribed by polymerase II, IV, and V; therefore, some may lack poly-A tails in plants^7^. There is increasing evidence suggesting that lncRNAs can fold into complex secondary and higher-order structures to provide greater potential and versatility for proteins and target recognition^4^,^8^,^9^. Therefore, lncRNAs may regulate protein-coding gene expression at the post-transcriptional and transcriptional levels. Emerging studies have revealed that lncRNAs are involved in diverse biological processes in mammals such as regulation of mating type, pluripotency of embryonic stem cells, apoptosis, organogenesis, and various diseases^8^,^10^. It is worth noting that some lncRNAs have also been characterized functionally in plant developmental processes and stress-responsive pathways^5^. For example, two well-studied lncRNAs are *COLD INDUCED LONG ANTISENSE INTRAGENIC RNA* (*COOLAIR*) and *COLD ASSISTED INTRONIC NONCODING RNA* (*COLDAIR*) from *Arabidopsis. COOLAIR* and *COLDAIR* regulate vernalization by interacting with the polycomb-responsive complex 2 (PRC2), further modulating vernalization-mediated epigenetic repression of the *FLOWERING LOCUS C* (*FLC*; a key flowering repressor in the vernalization pathway) and repressing *FLC* expression^11^. By solving the *in vitro* secondary structure of *COOLAIR*, Hawkes *et al*. found the distal *COOLAIR* transcript is highly structured in *Arabidopsis*, with numerous secondary structure motifs, an intricate multi-way junction, and two unusual asymmetric 5′ internal loops (right-and turn [r-turn] motifs)^12^. Interestingly, its secondary structure has been evolutionarily conserved across species despite low sequence conservation^12^. Recent work also discovered ASL (Antisense Long) transcript in early-flowering *Arabidopsis* ecotypes that do not require vernalization for flowering^13^. ASL is transcribed from the same promoter as *COOLAIR* and their 5′ regions partially overlap. Distinct from other lncRNAs at *FLC*, ASL lncRNA was shown to be involved in the regulation of the autonomous flowering pathway^13^. Another intergenic lncRNA called *INDUCED BY PHOSPHATE STARVATION1* (*IPS1*) has also been discovered in *Arabidopsis*, which is induced by phosphate starvation and acts as a decoy for miR399 to allow the accumulation of its target gene transcripts^14^,^15^. The lncRNA *LONG DAY SPECIFIC MALE FERTILITY ASSOCIATED RNA* (*LDMAR*) from rice may be an important player in regulating male development in response to environmental cues^16^. LncRNAs can also regulate intron splicing of the sense transcripts by masking splicing sites through its complementary sequences. For example, alternative splicing competitor lncRNA (ASCO-lncRNA) can hijack nuclear speckle RNA-binding protein (NSR) to alter splicing patterns of transcripts in response to auxin in *Arabidopsis*^17^. Recently, genome-wide discoveries for lncRNAs have been conducted across plants, such as *Arabidopsis, Triticum aestivum, Oryza sativa, Zea mays, Populus trichocarpa*, and *Fragaria vesca*^18^,^19^,^20^,^21^,^22^,^23^. Moreover, some important online databases of lncRNAs were also created, such as CANTATAdb, LncVar, and NONCODE^24^,^25^,^26^. To our knowledge, no studies have addressed the roles of lncRNAs in citrus, despite the great interest in their biological processes.

Citrus is one of the most widespread fruit crops globally, with tremendous economic and health values. Flowering is an essential stage for fruit production, and our understanding of the genetic mechanisms underlying the flowering event is critical for genetic improvements across plants. Citrus flowering has consistently been the goal of ongoing investigations; however, the long juvenile stage presents a major obstacle in traditional breeding and genetic studies of citrus. Precocious trifoliate orange (MT), an early flowering mutant of *Poncirus trifoliata*, has a shorter juvenile stage compared with its wild-type (WT) counterpart. Approximately 20–30% of seedlings germinate from MT seeds flowered during the first year after germination, whereas the WT usually has a juvenile period of 6 to 8 years^27^. Numerous studies have been conducted to decipher the molecular mechanism underlying the early flowering between MT and WT^28^,^29^,^30^. For example, a previous transcriptional study illustrated the differential expression of many genes associated with flowering processes between MT and WT and showed that *FLOWERING LOCUS T* (*FT*) transcripts accumulated to higher levels and *TERMINAL FLOWER1* (*TFL1*) transcripts accumulated to lower levels in MT relative to WT at the phase transition from the vegetative stage to the flowering stage in MT^30^. Additionally, many miRNAs involved in flowering development have been identified^28^,^31^. Recently, genome resequencing was also performed for MT and WT, and a large amount of differential genetic variation was detected^29^. However, the mechanism involved in the early flowering mutant remains essentially unknown. Therefore, it is necessary to identify novel lncRNAs and to understand the function of lncRNAs in citrus flowering.

In the present study, a comprehensive analysis of lncRNAs from MT and WT counterparts was performed using paired-end strand-specific RNA sequencing (ssRNA-Seq). A total of 6,584 putative lncRNAs were identified. Compared with WT, 831 lncRNAs showed significantly differential expression between MT and WT at the phase transition stage. Overall, our investigation revealed that lncRNAs can play a significant role in the response of trifoliate orange flowering. These findings also provided new insights for further research assessing the molecular mechanisms of lncRNAs and related miRNA pathways in citrus flowering.

## Results

A major characteristic of the MT is that the juvenile period is 1 to 2 years, whereas that of the WT is 6 to 8 years^27^. Previous studies showed that the stage of self-pruning for spring shoots is the critical stage for flower bud differentiation of MT^28^,^31^. Cytological observations revealed that the floral buds in MT initiated their differentiation immediately after self-pruning. However, the spring shoots of the WT do not form floral buds; instead, they begin to produce vegetative buds^28^,^31^. In this study, the ages of the MT and WT plants were similar when they were sampled. The floral buds in MT initiated differentiation at this stage. However, the WT did not form floral buds and began to produce vegetative buds. To identify flowering-related lncRNAs in trifoliate orange, paired-end ssRNA-seq of transcripts from MT and WT after the self-pruning stage of spring shoots were conducted in three biological replicates. More than 96 million raw reads were produced from each biological replicate after discarding low-quality reads, removing filtering 5′ contaminant, and trimming 3′ adaptor reads. The average read depth of this sequencing was approximately 175-fold that of the whole transcriptome (56.5 Mb). This large amount of data allowed the detection of both rare and species-specific transcripts in MT and WT. A total of 51,744 transcripts were assembled by RNA-Seq from the WT and MT.

**Figure 1:**
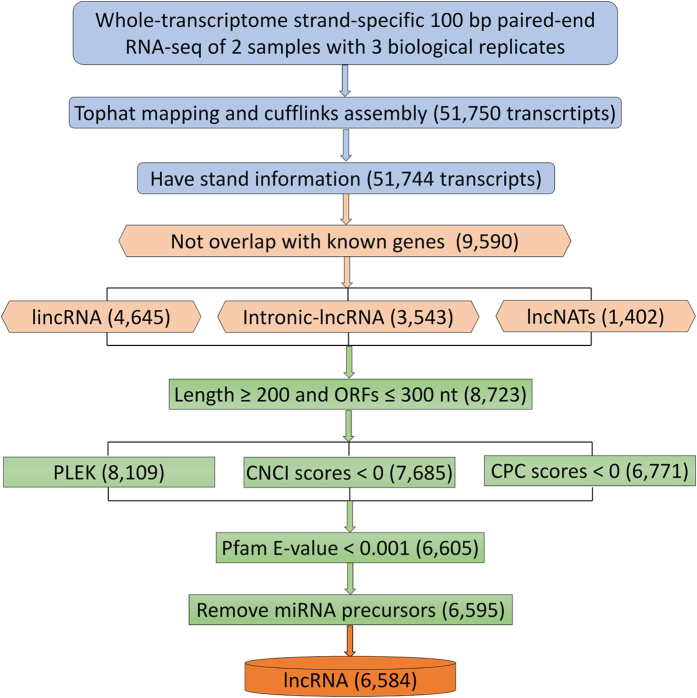
Detailed schematic diagram of the informatics pipeline for the identification of citrus lncRNAs. Paired-end strand-specific RNA-Seq was performed for MT and WT. Clean reads were mapped and assembled according to the known citrus genome using TopHat and Cufflinks^49^. Transcripts were filtered with the six criteria for identification of putative lncRNAs: (i) not citrus coding genes; (2) length >200 nucleotides and ORF < 100 amino acids; (iii) not encoding known protein domains; (iv) little coding potential; (v) not housekeeping ncRNAs; and (vi) not miRNA precursors. A total of 6,584 transcripts were obtained. CPC: coding potential calculator; PLEK: predictors of long non-coding RNAs and messenger RNAs based on an improved k-mer scheme; CNCI: coding non-coding index.

To distinguish potential lncRNAs, several sequential stringent filters were used for the 51,744 transcripts (Fig. 1). First, these transcripts were filtered with citrus coding gene sequences (http://www.phytozome.net/clementine.php). Almost 81.5% (42,154) of the transcripts were coding genes (the transcripts with significant alignment [P < 1.0E-10, identity >90%, coverage >80%] with citrus proteins were excluded); the remaining 18.5% (9,590) of transcripts might be non-coding RNA. It is generally believed that lncRNAs are at least 200 bp in length and do not encode for an ORF of more than 100 amino acids. This filter was then applied to the 9,590 transcripts; 8,723 transcripts were recovered. These transcripts were further filtered by comparing them with the four protein databases (KEGG, NR, COGs, and Swiss-Prot) to eliminate transcripts encoding conserved protein domains (Fig. 1). Next, the CPC was used to assess the protein-coding potential to eliminate possible coding transcripts. After using the four stringent criteria, 6,771 transcripts were considered putative lncRNAs. Because housekeeping ncRNA (tRNAs, snRNAs, and snoRNAs) and miRNA precursors are two specific species of lncRNAs that function differently from other lncRNAs, the putative lncRNAs were next aligned to comprehensive sets of housekeeping ncRNAs and miRNA precursor sequences, respectively. Thus, a total set of 6,584 transcripts was obtained (Table S1) based on the stringent sequential filters described (Fig. 1). To investigate the conservation of trifoliate orange lncRNAs, putative lncRNAs were aligned with lncRNAs from *Arabidopsis*, tomato, and *Populus trichocarpa*^20^,^22^,^26^. We could only detect two lncRNAs (TCONS_00043895 and TCONS_00050718) that were comparable to those lncRNAs in *Arabidopsis*.

### Distribution of lncRNAs in the citrus genome

**Figure 2:**
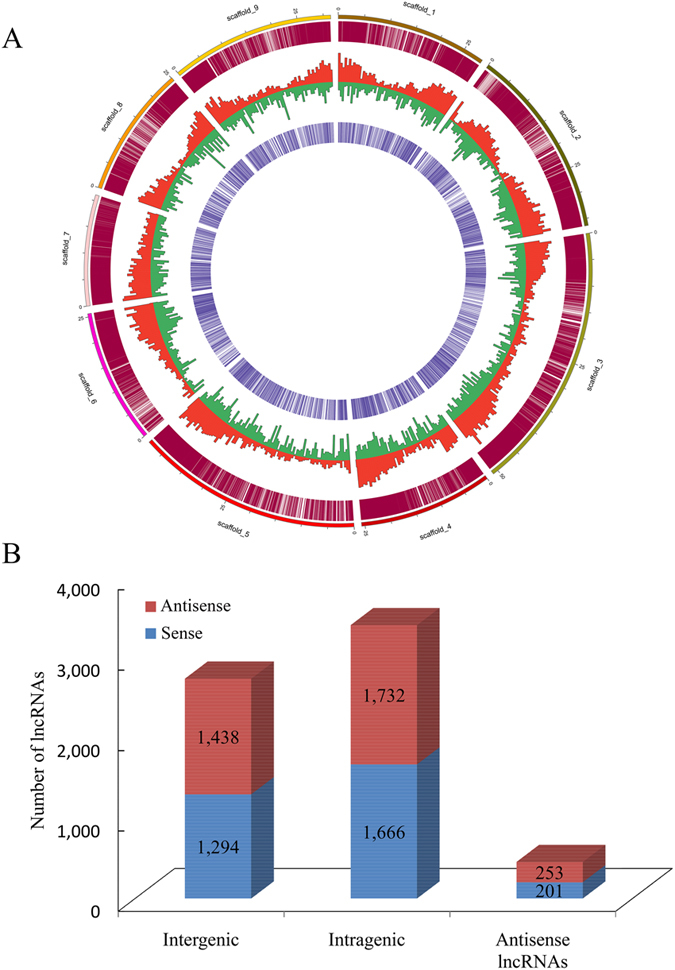
Distribution and classification of 6,584 citrus lncRNAs. (**A**) Genome-wide distribution of citrus lncRNAs compared with protein-coding genes. Chromosomes are indicated in different colors and in a circular form as the outer thick track. The inner chromosome scale (Mb) is labeled on each chromosome. On the second track (outer to inner), each vertical red line shows the location of protein-coding genes throughout the whole citrus genome. In the next two tracks, the abundances of protein-coding genes and lncRNAs in physical bins of 10 Mb per chromosome are indicated by blue and red columns, respectively. On the fourth track, each vertical purple line shows the location of lncRNAs throughout the entire citrus genome. (**B**) Classification of citrus lncRNAs according to their genomic position and overlap with protein-coding genes. Numbers of lncRNAs in the sense or antisense strand for each of the three main classes are labeled in the columns (intergenic, intragenic, and antisense).

The lncRNAs were mapped onto the recently released citrus nine scaffolds (equivalent to nine citrus chromosomes)^32^. The results showed that citrus lncRNAs have lower densities in the pericentromeric heterochromatin regions than in the euchromatin (Fig. 2A). However, most protein-coding genes (except protein-coding genes similar to lncRNAs in scaffold 2) were evenly distributed on eight chromosomes. These results suggest that lncRNAs might have different transcriptional features than the protein-coding genes in citrus (Fig. 2A). In addition, some lncRNAs have been transcribed for loci much closer to the telomeres than protein-coding genes. For example, some lncRNAs were generated from the ends of scaffolds 5 and 6 (Fig. 2A). According to the locations relative to the nearest protein-coding genes, lncRNAs were further classified into three types: lncRNAs without any overlaps with any protein-coding genes (intergenic lncRNAs), lncRNAs totally in the some protein-coding loci (intragenic lncRNAs), and lncRNAs with exonic overlaps with any exons of protein-coding genes on the opposite strand (antisense lncRNAs). Although 6.9% and 41.5% of the lncRNAs either were antisense lncRNAs or were transcribed from within genes (most from introns), the majority of lncRNAs (51.6%) were located in intergenic regions (Fig. 1B). Interestingly, the numbers of the three types of lncRNAs between sense and antisense strands were similar.

### Characterization of trifoliate orange lncRNAs

**Figure 3:**
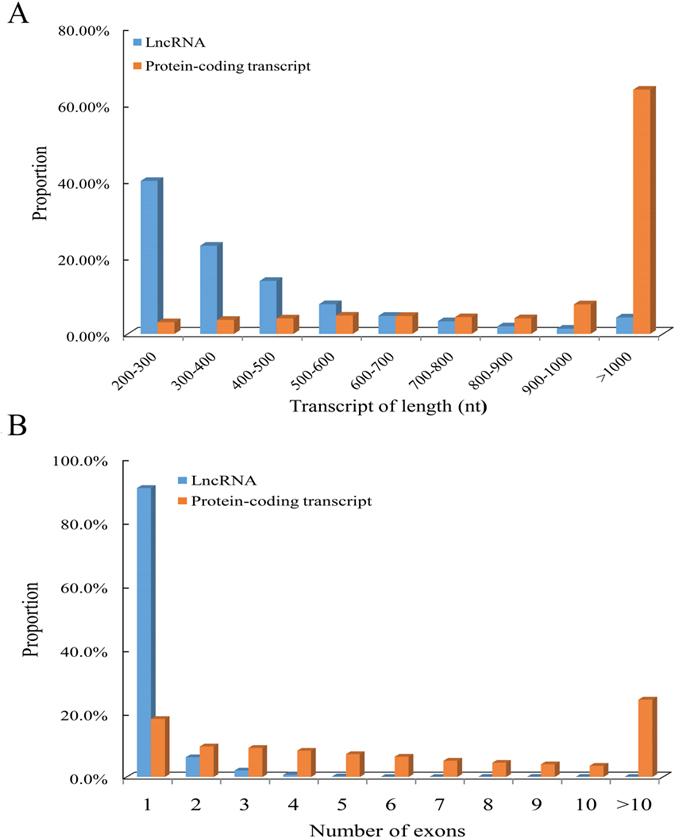
LncRNAs are shorter and have fewer exons than protein-coding transcripts. Distribution of the length (**A**) and numbers of exons (**B**) for the 6,584 lncRNAs in comparison to the 33,929 protein-coding transcripts in citrus.

The lncRNAs in plants have been reported to be shorter and to consist of fewer exons compared with protein-coding genes^5^. Therefore, the distribution of the length and exon number of the 6,584 lncRNAs was analyzed compared with all predicted protein-coding transcripts in citrus (33,929 transcripts in the reference genome). The results indicated that the distribution of the length of these lncRNA ranged from 200 bp to 4,026 bp, which is approximately 95.8% of the lncRNAs ranging in size from 200 to 1,000 bp, with only 4.2% having a size >1,000 bp; the most abundant lengths ranged from 200 to 400 bp (Fig. 3A). In contrast, approximately 63.8% of the protein-coding transcripts were >1,000 bp. Remarkably, most of the genes (96.9%) encoding trifoliate orange lncRNAs contained only one or two exons, whereas the number of exons in the protein-coding genes ranged from 1 to ≥10 (Fig. 3B). These results indicate that the majority of the trifoliate orange lncRNAs are relatively shorter and contain fewer exons compared with protein-coding genes.

### Identification of flowering-related lncRNAs

**Figure 4:**
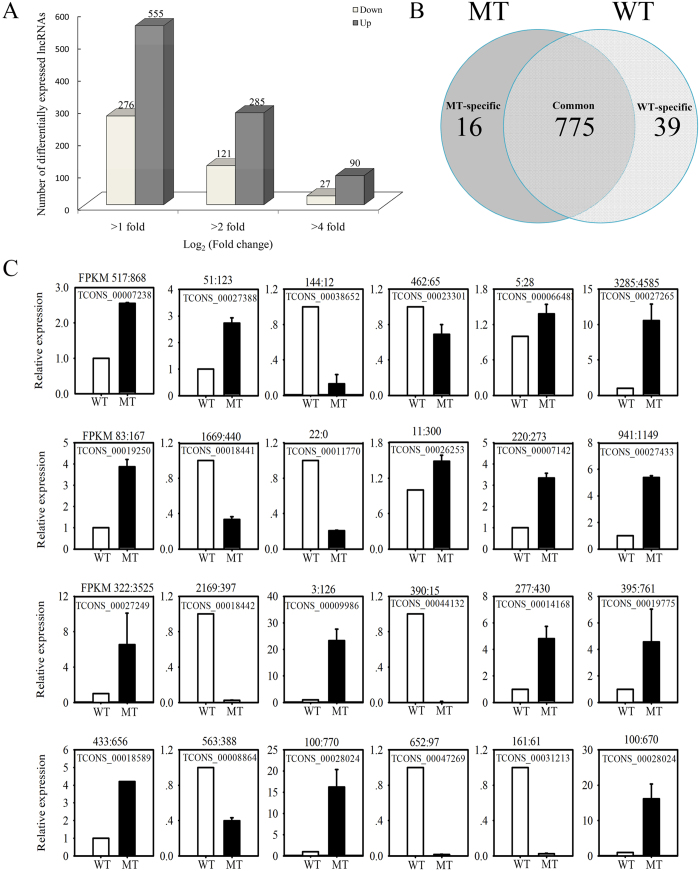
Differential expression of flowering related lncRNAs. (**A**) Total numbers of differentially expressed lncRNAs (|log_2_ ratio| ≥ 1, 2, and 6; *P* < 0.05) between MT and WT. (**B**) Venn diagram showing the differentially expressed lncRNAs between MT and WT. (**C**) Real-time PCR validation of RNA-Seq data showing the accumulation of 24 randomly selected lncRNAs between WT (white columns) and MT (black columns); the abundance of lncRNAs from RNA-seq data is shown above each lncRNA. Relative transcript levels are calculated by real-time PCR with β-actin as the standard. Data are means ± SE of four separate measurements.

To identify flowering-related lncRNAs in trifoliate orange, the lncRNA expression values of MT and WT were calculated (FPKM units) and compared. The lncRNAs were differentially expressed between MT and WT based on *P* < 0.05 and an absolute value of log_2_ ratio ≥1 as a threshold. A total of 831 lncRNAs were significantly differentially expressed between MT and WT (Fig. 4A). Compared with MT, 555 of 831 lncRNAs were up-regulated in MT and the other 276 lncRNAs were down-regulated (Table S2). Of these, 16 were not observed in the WT library and 39 were not present in the MT library (Fig. 4B). To investigate whether these differentially expressed lncRNAs are involved in flowering, 24 of them were arbitrarily selected (13 from up-regulated, 8 from down-regulated, and 3 from no differential expression). The differences in expression levels observed by RNA-Seq were experimentally validated by real-time polymerase chain reaction (PCR) (Fig. 4C). The lncRNA abundance patterns of MT and WT were compared with the RNA-Seq results. The results showed that for 20 of the 24 genes, real-time PCR revealed the same expression tendency as the RNA-Seq data, despite some quantitative differences in expression levels. Figure 4 shows the differential expression levels for 24 genes between MT and WT.

### Differential expression of flowering-related protein-coding genes between MT and WT

**Figure 5:**
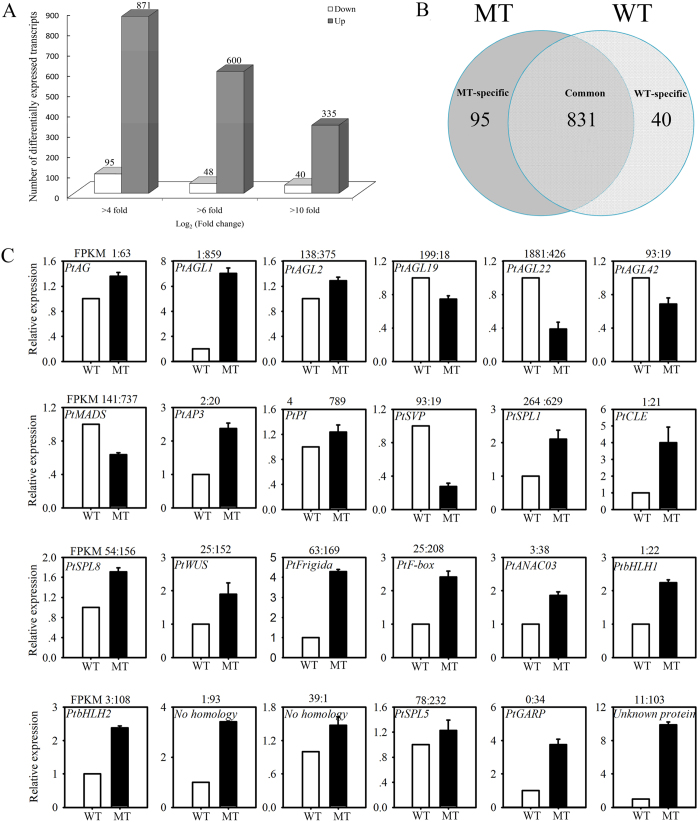
Differential expression of protein-coding genes. (**A**) The total numbers of differentially expressed transcripts (|log_2_ ratio| ≥ 4, 6, and 10; *P* < 0.05) between MT and WT. (**B**) Venn diagram showing the differentially expressed transcripts between MT and WT. (**C**) Real-time PCR validation of RNA-Seq data showing the accumulation of 24 randomly selected flowering-related transcripts between WT (white columns) and MT (black columns); the abundance of protein-coding genes from RNA-seq data is shown above each gene. Relative transcript levels are calculated by real-time PCR with β-actin as the standard. Data are means ± SE of four separate measurements. The gene ID from the citrus genome database (https://phytozome.jgi.doe.gov/pz/portal.html#!info?alias=Org_Cclementina).

We detected the expression of 29,951 read-mapped protein-coding genes in MT and WT, and the expression levels of these genes are also measured by RPKM. Among them, 14,264 genes were down-regulated and 15,687 genes were up-regulated in MT compared with WT. Nine hundred sixty-six genes were differentially expressed between MT and WT based on *P* < 0.05 and an absolute value of log_2_ ratio ≥4 as a threshold (Fig. 5A). Of these, 871 were more abundant and 95 were less abundant in MT compared with WT, suggesting that many genes were enriched during the flowering transition. A total of 831 differentially expressed genes were common to both MT and WT, and only 95 and 40 genes were expressed specifically in MT and WT, respectively (Fig. 5B). BLAST searches of the 966 genes showed that many genes had a high identity with known transcription and post-transcriptional regulatory genes, indicating that these genes may be key regulators controlling flower development by activating or repressing numerous protein-coding genes (Table S3). Moreover, a number of transcription factors including MYB and MADS-box were observed, which have been implicated in flower development and flowering time^33^,^34^. Additionally, some differently expressed genes were involved in transcription, chromatin remodeling, hormone regulation, and other metabolic pathways (Table S3).

To validate the expression profiles obtained using RNA-Seq, real-time PCR was performed using 24 differentially expressed genes that were selected based on high or low expression levels (including 10 MADS transcription factors, three *SQUAMOSA PROMOTER BINDING PROTEIN-LIKE* [*SPL*] transcription factors, two basic helix-loop-helix DNA-binding transcription factors, six protein-coding genes involved in flowering, one unknown protein, and two genes without matches in the database). The results showed that for 22 of the 24 genes, real-time PCR revealed the same expression patterns as the RNA-Seq results, despite some quantitative differences in expression levels (Fig. 5C).

### Predicted interactions between miRNAs and lncRNAs

A recent report indicated that lncRNAs could also be targeted by miRNAs in plants^20^. In previous reports, a larger number of conserved and trifoliate orange–specific miRNAs were identified in WT and MT^28^,^31^. To systematically investigate the miRNA-mediated regulatory mechanism of lncRNAs in trifoliate orange, psRobot was applied to predict miRNA targets among 6,584 lncRNAs. Among 141 conserved and 102 trifoliate orange-specific miRNAs, 58 miRNA-lncRNA interactions were found. A total of 23 targets of 14 conserved miRNAs in 10 families were identified, including a series of targets of conserved miRNA families including Pt-miR156, Pt-miR172, and Pt-miR396 (Table S4). It is noteworthy that a total of 26 target genes were also identified for 20 trifoliate orange-specific miRNAs (Table S5). A total of 27 miRNAs were predicted to target the antisense strand of lncRNAs, whereas 22 were also found to target the sense strand. Most miRNAs, especially the conserved ones, could target several genes; for example, Pt-miR172a, Pt-miR172e, and Pt-miR396a had at least three targets. The trifoliate orange-specific miRNAs appeared to have only a limited number of targets, excluding Nove129, Nove135, Nove136, Nove142, and Pt-miR243 (the miRNA was named according to previous reports)^28^,^31^. A total of two and six potential lncRNAs were predicted for targets of the miR156 and miR172 families, respectively (Table S4).

The lncRNAs that potentially function as target mimics of miRNAs were predicted according to Wu^35^. In total, seven lncRNAs were identified that may act as target mimics and may be bound by nine miRNAs (three known miRNAs and six novel miRNAs) to form nine miRNA-lncRNA duplexes (Table S6). Among these miRNA target mimics, TCONS_00030665 was the target mimic of Pt-miR156a; TCONS_00041402 was the target mimic of Pt-miR160a. It is worth noting that TCONS_00041402 is the target mimic of one known miRNA (Pt-miR160a) and two novel miRNAs (Nove171 and Nove125) (Table S6).

### Monitoring the expression of potential targets of miRNAs

**Figure 6:**
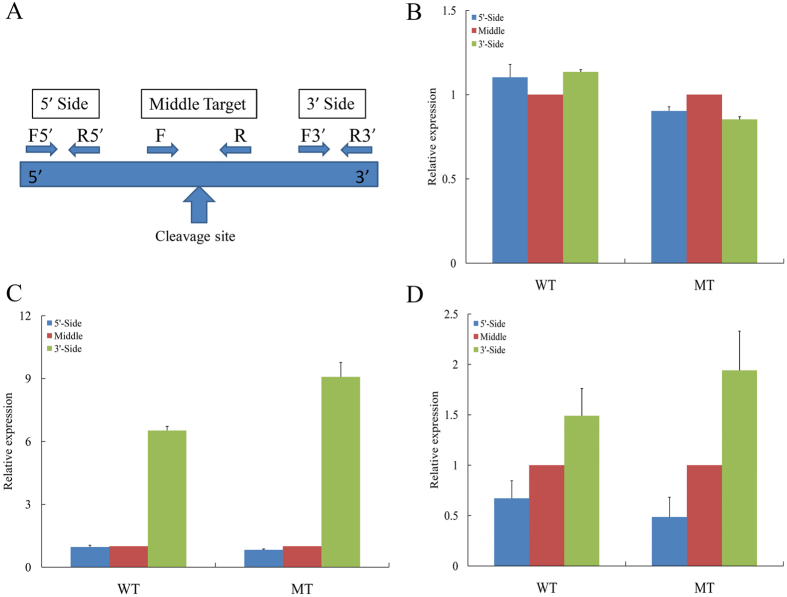
Regional amplification quantitative RT-PCR (RA-PCR) of the targets. (**A**) Diagram of the primers designed to amplify fragments of cleaved and non-cleaved miRNA-targeted lncRNAs and miRNA target sites. (**B**) Relative quantification of three fragments of the control (TCONS_00048785) by RA-PCR, with the middle target fragment set to a value of 1. (**C**) Relative quantification of three fragments of TCONS_00030665 by RA-PCR, with the middle target fragment being set to a value of 1. (**D**) Relative quantification of three fragments of TCONS_00004802 by RA-PCR, with the middle target fragment being set to a value of 1. Relative transcript levels are calculated by real-time PCR with β-actin as the standard. Data are means ± SE of four separate measurements.

Previous studies of mRNAs with miRNA target sites suggest that only the 3′ fragment of the target mRNA possesses a poly(A) tail after miRNA-induced cleavage^36^. When poly (T) adapters are used for reverse transcription, only the 3′ end of the cleaved mRNA should be copied into cDNA. Therefore, the reverse-transcription PCR (RT-PCR) product from the upstream region of the cleaved site is no greater than the downstream region because reverse transcription with poly (T) adapters would not generate cDNA beyond a cleaved site. Here, to verify the miRNA targets, regional amplification quantitative RT-PCR (RA-PCR)^36^ was used to assess the abundance of three regions of potential targets of miRNAs based on the aforementioned method. Figure 6A illustrates the relative positions of the three primer sets for a given lncRNA for the RA-PCR method. The first set (F5′, R5′) amplifies a region upstream of the potential cleavage site. The second set (F, R) synthesizes a fragment that contains the cleavage site and flanking regions. The third set (F3′, R3′) amplifies a fragment downstream of the cleavage site. Primers were tested to ensure amplification of single discrete bands with no primer–dimers, additional primer-binding sites, and self-priming caused by RNA secondary structures.

Among these 49 targets and 9 target mimics, 21 lncRNAs were investigated using the RA-PCR method. Figure 6B shows the results of the RA-PCR for TCONS_00048785 in MT and WT. This lncRNA does not have a known miRNA target site and was used as a negative control. The results indicate that the stability of the middle region is similar to that of the 3′ and 5′ regions for this lncRNA. The PCR products from the three regions of most of the lncRNAs exhibited similar levels for both MT and WT. Differences were noted for these regions in TCONS_00030665, meriting more detailed analyses. Figure 6C shows the products of the RA-PCR reactions for the expression of TCONS_00030665, a potential target of Pt-miR156 during the phase transition stage of the two genotypes. The middle and 5′ regions were decreased relative to the 3′ regions; the amount of product from the 3′ region was 6.5-times to 9.2-times the amount observed from the 5′ and middle regions in MT and WT, respectively, but was similar to the amount observed from the 5′ and middle regions. This finding is consistent with miRNA-induced cleavage of this mRNA. Figure 6D shows the results from RA-PCR analysis of the three regions amplified from TCONS_00004802, a potential target of Pt-miR396a from the juvenile to the adult stage of the two genotypes. The results indicated that the target region also decreased relative to the 3′ regions. It is noteworthy that the 5′ region appeared to decay faster than the 3′ region. The amount of product from the 5′ region was 0.48-times to 0.67-times the amount observed from the middle region. This result may be due to a preferential decay from the 5′ side^37^. This is, again, in agreement with the miRNA-induced cleavage of this mRNA. These results indicate that TCONS_00030665 and TCONS_00004802 may be regulated by Pt-miR156a and Pt-miR396a, respectively.

### Tissue specificity of lncRNA expression in MT and WT

**Figure 7:**
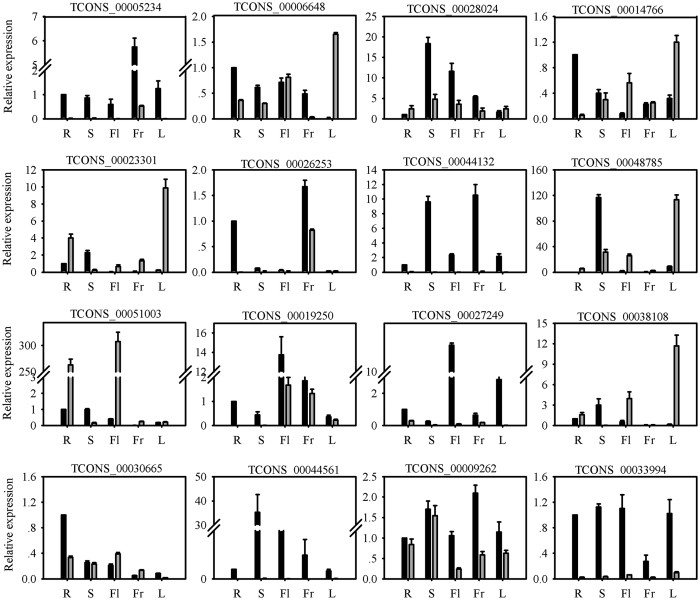
Relative quantities of lncRNAs in various tissues from MT (gray column) and WT (black column): roots, shoots, leaves, flowers at anthesis, and whole fruits at 30 days after flowering. Relative transcript levels are calculated by real-time PCR with β-actin as the standard. Data are means ± SE of four separate measurements.

A major challenge in predicting lncRNA function resides in the lack of conservation. Their expression patterns, specifically tissue-specific expression patterns, may aid in deducing the potential function of these lncRNAs. Therefore, to examine the expression patterns of flowering-related lncRNAs in more detail, the expression pattern of 16 lncRNAs (11 differentially expressed lncRNAs and 5 targets of miRNAs) were analyzed by real-time PCR in roots, spring flushes, leaves, flowers at anthesis, and whole fruits at 30 days after flowering (Fig. 7). These lncRNAs, excluding TCONS_00044561, could be detected in roots, spring flushes, leaves, flowers, and fruit by real-time PCR. However, most lncRNAs were present at low levels in MT tissue compared with the levels in WT tissue. TCONS_00006648, TCONS_00028024, TCONS_00014766, TCONS_00030665, and TCONS_00009262 showed broad expression patterns, with transcripts detected in all plant organs in MT and WT. TCONS_00019250 and TCONS_00027249 were expressed at higher levels in flowers than in the other organs. TCONS_00051003 was expressed predominantly in leaves and flowers. TCONS_00030665 exhibited the highest expression levels in roots. TCONS_00023301 and TCONS_00038108 were expressed mainly in leaves, and TCONS_00005234 and TCONS_00026253 showed higher expression levels in fruit and spring flushes. Five lncRNAs were specific to WT (TCONS_00005234, TCONS_00044132, TCONS_00027249, TCONS_00044561, and TCONS_00033994) and two lncRNAs were specific to MT (TCONS_00051003 and TCONS_00038108). It is noteworthy that three targets (TCONS_00044561, TCONS_00033994, and TCONS_00038108) were specific to WT or MT. These results indicated that the three lncRNAs may be regulated by miRNAs during the flowering development processes.

### Functional analysis of Pt-miR156 and its target

**Figure 8:**
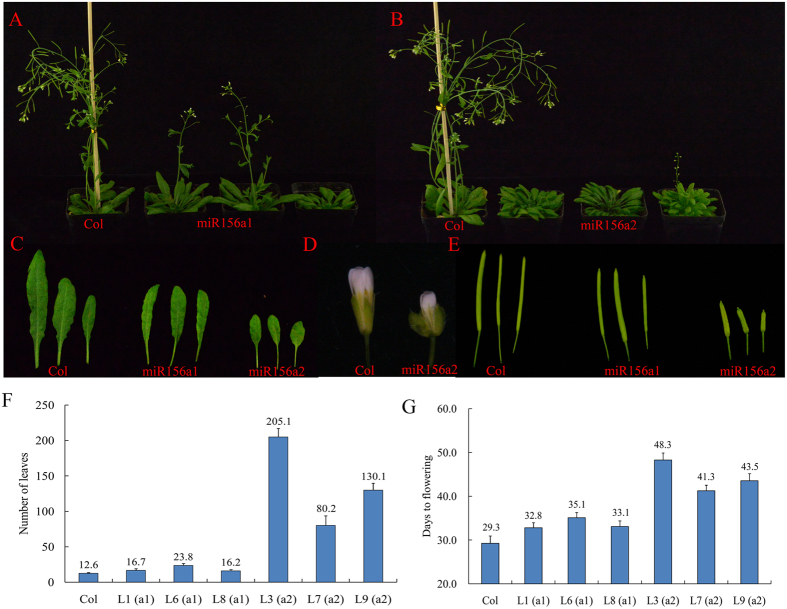
Overexpression of Pt-miR156a1/a2 and phenotypic analysis in *Arabidopsis*. (**A**) Phenotypes of transgenic *Arabidopsis* with p35S:Pt-miR156a1 under long day. (**B**) Phenotypes of transgenic *Arabidopsis* with p35S:Pt-miR156a2 under long day. (**C**) Leaves of p35S:Pt-miR156a1 and p35S:Pt-miR156a2 transgenic and control plants. (**D**) Flower phenotypes of control and p35S:Pt-miR156a2 plants. (**E**) Silique length of control and p35S:Pt-miR156a1/a2 transgenic *Arabidop*sis. (**F**) Times to flowering of T_3_ plants of six independent transgenic lines from Pt-miR156a1 (L1, L6, and L8) and Pt-miR156a2 (L3, L7, and L9). (**E**) Number of leaves to flowering of T_3_ plants of six independent transgenic lines from Pt-miR156a1 (L1, L6, and L8) and Pt-miR156a2 (L3, L7, and L9).

The miR156 family plays an important role in regulating the vegetative phase change of *Arabidopsis*. TCONS_00030665 may be regulated by Pt-miR156a based on RA-PCR analysis. These results indicated that TCONS_00030665 might be also important for the flowering of trifoliate orange. In addition, the miR156 family is highly conserved between citrus and *Arabidopsis*^31^. Genetic transformation of citrus is very difficult because of a variety of technical limitations associated with citrus. Thus, to characterize the functions of miR156 and its targets, three expression constructs containing the Pt-miR156a1/2 precursors and one lncRNA (TCONS_00030665) under the control of the 35S promoter were genetically transformed into *Arabidopsis* (Fig. 8). Twenty-three and 21 independent Kanamycin-resistant plants were obtained in the T_1_ generation for Pt-miR156a1 and Pt-miR156a2, respectively. PCR analysis showed that the precursor of Pt-miR156a1/a2 was ectopically expressed in transgenic lines but not in the control plants. Three independent transgenic lines from the T_3_ generation were randomly selected for phenotypic observation for each miRNA. Compared with the control plants, three transgenic lines from the 35S::Pt-miR156a1 transgenic lines showed slightly late flowering (Student’s *t* test, *P* > 0.05) in terms of both days to flowering and number of leaves (Fig. 8A and F). No differences in the appearance of flowers and inflorescences were observed among 35S::Pt-miR156a1 and the control. However, three transgenic lines from Pt-miR156a2 flowered significantly later than the control. The average time to flowering in the transgenic plants ranged from 43.5 to 48.3 days, whereas that in the control plants was 29.3 days. The average number of leaves at the time of flowering ranged from 80.2 to 205.1 in the transgenic plants and was 12.6 in the control plants. Interestingly, when the control plants began to senesce, the 35S::Pt-miR156a2 transgenic plants maintained vegetative growth and even failed to bolt (Fig. S1). In addition, the transgenic plants of Pt-miR156a2 showed multiple morphological changes, such as severe dwarfism, smaller leaves, and flowers under long days. It is noteworthy that the 35S::Pt-miR156a2 plants produced shorter and fewer siliques than control plants. The transgenic siliques were, on average, 50–60% as long as those of the control plants.

To determine the effects of the target of Pt-miR156 on flowering time and inflorescence morphology, TCONS_00030665 was also introduced into *Arabidopsis*. Twenty independent Kanamycin-resistant plants were obtained in the T_1_ generation. Using real-time PCR, CONS_00030665 was detected in transgenic plants and no transcript was detected in the control. However, TCONS_00030665 did not affect the timing of flowers or inflorescences in transgenic *Arabidopsis*.

## Discussion

Recent advances in RNA-Seq, which has been combined with genome-wide mapping, have resulted in the identification of a set of lncRNAs in plants^18^,^20^,^22^. These identified lncRNAs have opened up a new field in the investigation of novel regulatory pathways in plants^18^. For example, 3,679 lncRNAs from the tomato ripening inhibitor (*rin*) mutant and its WT counterpart have been identified, indicating that lncRNAs may induce an obvious delay in fruit ripening^22^. Zhang *et al*. (2014) identified a large number of lncRNAs involved in sexual reproduction of rice that were expressed in a tissue-specific or stage-specific manner^19^. In the present study, we investigated transcriptomic changes during the phase transition of precocious trifoliate orange and its WT counterpart and systematically identified the lncRNAs associated with flowering (Table S1). Moreover, we also identified several lncRNAs that were specifically or differentially expressed in the two genotypes. To date, although many lncRNAs have been identified in model plants such as *Arabidopsis*, tomato, *Populus trichocarpa*, rice, and maize, much work remains to be performed with citrus^19^,^20^,^21^,^38^. Here, we present the first comprehensive analysis of lncRNAs in trifoliate orange. These lncRNAs will probably be very useful for other citrus researchers and provide a useful resource for future functional genomics studies and regulatory expression research.

Trifoliate orange lncRNAs are not well-conserved compared with protein-coding genes, have fewer exons, are expressed at lower levels, and are shorter in transcript length, consistent with other studies examining plant lncRNAs^22^,^39^. In this study, most trifoliate orange lncRNAs (90.7%) contained only one exon; one possible reason is that the number of exons is not filtered in novel transcripts. However, if single-exon transcripts are removed from the ab initio transcriptome assemblies, then some of the real lncRNA may be lost. Using real-time PCR analysis, we also found that many lncRNAs have more specific expression profiles than coding genes. This finding is consistent with previous studies in which lncRNAs have highly specific temporal and spatial expression profiles^39^. However, it is noteworthy that only two homologues lncRNAs were identified in trifoliate orange compared with *Arabidopsis* in the NONCODE database^26^. These results suggest that the majority of lncRNAs identified in our study were not conserved with currently known lncRNAs among different plant species. Similar results were observed for lncRNAs in other plant species, such as *Populus*, tomato, maize, and wheat^20^,^21^,^22^,^23^. However, thousands of conserved lncRNAs have been found in mammals, possibly because of a more ancient origin of these lncRNAs and more time to acquire stabilized functions^40^,^41^. The low conservation suggested that these plant lncRNAs may undergo rapid evolution. In fact, it is not surprising that lncRNAs are not well-conserved because of several possible explanations. First, lncRNAs are not constrained by codon usage. Second, although lncRNAs may possess short conserved motifs, these short motifs are not easily identifiable by BLAST^39^. Third, some lncRNAs may directly interact with RNA-binding proteins through conserved secondary structures^18^.

Flowering is one of the most important adaptive traits to ensure the transition of reproductive development during a plant’s life cycle^42^. Several flowering regulatory pathways have been identified to date, such as the vernalization, photoperiod, circadian clock, age, and gibberellin pathways^42^. We also identified a larger number of differentially expressed protein-coding genes representing putative homologs to flowering-related genes in this study, consistent with our previous results from cDNA macroarray in combination with suppression subtraction hybridization^30^. Some of these genes are required for the day-length response and some encode regulatory proteins specifically involved in the control of flowering, whereas others encode components of light signal transduction pathways or are involved in circadian clock function. Vernalization is known to involve lncRNAs, primarily in the regulation of *FLC*^11^,^43^. *FLC* is located at a complex locus in the *Arabidopsis* genome. Recent studies have shown that at least two types of lncRNAs are present in this locus: *COOLAIR* and *COLDAIR. COOLAIR* is transcribed in the antisense orientation relative to *FLC*, whereas *COLDAIR* is transcribed from the intron of *FLC* in the sense orientation^11^. Both lncRNAs can help recruit the PHD-PRC2 complex to enable histone modifications of *FLC* via epigenetic regulation^43^. However, no significant matches were obtained with *COOLAIR* and *COLDAIR* among the 6,584 lncRNAs in this study. One possible explanation for this observation is that the regulatory mechanism of *FLC* differs between *Arabidopsis* and trifoliate orange. A previous study indicated that the *FLC* homologue (*PtFLC*) is transcriptionally regulated by seasonal temperature fluctuations in trifoliate orange. It is regulated post-transcriptionally by alternative splicing via exon skipping, resulting in five splice variants^44^. The alternative splicing pattern of *PtFLC* is altered through developmental stages and further influenced by temperature fluctuations in trifoliate orange^44^. These findings suggest complicated regulation mechanisms in flower formation and flowering in perennial woody plants compared with model plants.

In contrast to our understanding of small ncRNAs, little is known about the functions and regulatory mechanisms of lncRNAs^2^. One intriguing mechanism is lncRNA-miRNA cross-talk^4^. Recently, some lncRNAs were identified as putative targets of miRNAs^20^,^22^; however, the evidence for lncRNA functioning miRNA targets is still lacking in plants. Previous RNA-Seq between MT and WT revealed the presence of a large number of miRNAs that are involved in the regulation of early flowering in MT^28^,^31^. Furthermore, degradome sequencing of MT revealed that a number of miRNA targets were genes that were previously already characterized as important factors during phase transition in MT^31^. For example, the target of miR156 is the *SPL* gene, which not only acts downstream of FT/FD but also defines a separate endogenous flowering pathway^42^. The target of miR172 is *APETALA2* (*AP2*), which is a negative regulator of citrus. In this study, a total of nine potential lncRNAs were predicted for targets of miR156/172. These results indicated that some lncRNAs may be involved in trifoliate orange flowering via the miR156/172 pathway. Transgenic *Arabidopsis* ectopically expressing Pt-miR156 exhibited more rosette leaves, delayed flowering, and dwarfism in our study. Considering the importance of miRNAs in the integration of flowering signals, the RA-PCR technique for monitoring miRNA-guided cleavage of lncRNAs was performed and the results indicated that two lncRNAs (TCONS_00030665 and TCONS_00004802) may be regulated by Pt-miR156 and Pt-miR396. However, ectopic expression of TCONS_00030665 and TCONS_00004802 did not affect the flowering time or flower development in transgenic *Arabidopsis*. There might be two possible explanations. At first, trifoliate orange lncRNAs are not conserved with currently known *Arabidopsis* lncRNAs. Therefore, the lncRNAs regulation mechanisms in seasonal responses and flower formation also may not be conserved between perennial woody plants and model plants. Second, these two lncRNAs may be regulated by multiple pathways besides the miRNA pathway, and these regulatory pathways also may not be conserved between citrus and *Arabidopsis*. Therefore, no significant differences were observed between WT and transgenic plants of different stages based on these reasons. Recently, lncRNAs were also observed to function as target mimics for some miRNAs, providing a new mechanism for the regulation of miRNA activity in plants^15^,^35^. Here, only seven target mimics from lncRNAs were predicted, indicating that target mimicry may not be a main function of lncRNAs in trifoliate orange. In this study, 58 targets were found among 6,584 lncRNAs. This number is lower than that previously reported for *Populus trichocarpa* (71)^20^. However, the number is significantly higher compared with reports involving the tomato (six)^22^. These results may reflect underlying differences in the regulatory mechanism of lncRNAs among different species.

Although the function of most lncRNAs remains unknown, evidence suggests that lncRNAs may play regulatory roles by interacting with RNA, DNA, and protein-coding genes^38^. For example, it has been reported that lncRNAs can act either in *cis* or in *trans* to regulate protein-coding gene expression in animals and plants^11^. The discovery of lncRNAs laid the groundwork for future functional studies of lncRNAs in trifoliate orange. Therefore, to understand the biological role of lncRNAs and their regulatory mechanisms in woody plants, future research should include functional analyses of these genes using overexpression or RNA interference gene silencing strategies in trifoliate orange to elucidate their specific roles.

## Methods

### Plant material

Tissues from MT and WT were collected from the experimental fields of the National Citrus Breeding Center (30°28′N, 114°21′E, 30 miles above sea level) at Huazhong Agricultural University. A major characteristic of MT is that its juvenile phase is shortened to 1 to 2 years, whereas the WT plant has a long juvenile period of 6 to 8 years^27^. Therefore, the WT plants did not form floral buds at similar ages. Self-pruning is a physiologic phenomenon in citrus in which shoots cease vegetative growth by automatically withering the shoot tip (0.5–1 cm). Self-pruning is a necessary but insufficient condition for floral bud initiation^45^. In this study, the seeds of WT and MT were planted in 20-cm pots containing a potting mix of a commercial medium and perlite at a ratio of 3:1 in January. The juvenile potted seedlings were then transplanted and grown under field conditions during the growing season. These juvenile trees were watered regularly with a nutrient solution. Until the late stage of self-pruning, the shoot apical meristem of juvenile trifoliate orange (including MT and WT) is in an undetermined state and floral primordia are not observed (October). After self-pruning, the new terminal bud and lateral buds of the juvenile trees enter dormancy until late February of the next year, and these buds develop into spring shoots in late winter or early spring of the next year. The spring flush is the most important one for growth and flower formation^46^. Cytological observation revealed that the floral buds in MT initiated their differentiation immediately after self-pruning on spring shoots. For WT, the spring shoots, which did not form floral buds, began to produce vegetative buds after self-pruning. Therefore, this period is called the phase transition stage of MT. In this experiment, the terminal bud and the five following buds (the major node position for flower formation) from spring flushes of these MT and WT trees were collected after self-pruning every 2 days and every 1 week, respectively. To prepare a representative sample of total RNA from the WT and MT tissues for RNA sequencing, different developmental stages of plant organs from approximately equal numbers of WT and MT were pooled for each plant type. For spatial expression analysis of lncRNAs, terminal meristems of spring flushes, leaves, roots, flowers at full bloom, and whole fruits at 30 days after flowering of MT and WT were also collected. All materials were collected from three individual plants, immediately frozen in liquid nitrogen, and stored at −80 °C until analysis.

### Total RNA isolation and paired-end strand-specific RNA sequencing

Total RNA was isolated from WT and MT (three biological replicates per genotype) using the Plant RNAiso Plus according to the manufacturer’s instructions (Takara, Kusatsu, Japan). Genomic DNA was removed from total RNA by DNase treatment (Promega, Madison, WI, USA). All RNA samples were quantified and examined for protein contamination and reagent contamination using a NanoDrop ND 1000 spectrophotometer (NanoDrop, Wilmington, DE, USA). Due to some lncRNAs lacking the poly(A) tail, the rRNA of total RNA was removed using Ribo-Zero rRNA Removal Kits (Plant) according to the manufacturer’s instructions (Illumina, USA), retaining lncRNA both with and without a poly(A) tail. Six strand-specific RNA libraries with an insert size of approximately 250 to 500 nucleotides were prepared according to a UTP method^47^ and submitted to the Beijing Genomics Institute (BGI, Shenzhen, China) for 125-bp paired-end sequencing on the Illumina HiSeq 2500 at a depth of approximately 90 million reads per library. The RNA-Seq data from this study have been submitted to Gene Expression Omnibus under accession number GSE84443.

### Bioinformatics analysis to identify lncRNAs

The paired-end RNA-Seq reads were filtered for quality using the FASTX-Toolkit^48^ with default parameters by removing low-quality reads and adaptor sequences. The clean reads from each library were aligned with the citrus reference genome^32^ using TopHat^49^. Only reads with no more than two mismatches were obtained and used to construct transcripts of each sample separately using Cufflinks^49^ based on the citrus genome reference. The lncRNA structure was optimized according to the read distribution, information of paired-ends, and the genome annotation. The distribution of reads in the genome was obtained by aligning the continuous and overlapping reads to form a transcription active region (TAR). The TARs were identified based on the method described by Shuai^20^. After the TARs were obtained, they were subjected to some filtering processes to identify lncRNA candidates^20^. First, the length of the TARs had to be longer than 200 bp to exclude small intergenic transcripts. Second, the longest ORF of the TAR had to be smaller than 100 amino acids (the longest ORF predicted by OrfPredictor)^50^. Both strands of the TARs were used for prediction. Third, to ensure that our results were not influenced by genomic DNA contamination of the cDNA library, the lncRNA candidates had to appear in both MT and WT. Fourth, the coding potential of the remaining transcripts was evaluated using coding potential calculator (CPC) software^51^, predictors of long non-coding RNAs and messenger RNAs based on an improved k-mer scheme (PLEK) software^52^, and Coding Noncoding Index (CNCI) software^53^. When applying CPC, we used the protein-coding transcripts of citrus as a reference. All transcripts with CPC scores >0 or CNCI >0 were discarded. Additionally, by searching against the Pfam database (http://pfam.xfam.org/, E-value < 0.001)^54^, transcripts encoding any conserved protein domains were removed from the sense strand for multi-exonic transcripts or from either strand for single exon transcripts. The Rfam database was used to discard housekeeping RNAs, such as tRNAs, rRNAs, snRNAs, and snoRNAs (E-value < 0.001) with BLASTN. Finally, these sequences were further analyzed using BLAST analysis against miRbase to remove miRNA precursors.

### Localization of lncRNAs and classification of lncRNAs

A diagram was generated to show the localization and abundance of lncRNAs and protein-coding genes in the citrus genome using the program Circos^55^. The annotated lncRNAs were subdivided into three categories according to the locations relative to the nearest protein-coding genes using the cuffcompare program in the Cufflinks suite^49^,^56^: (i) lncRNAs without any overlap with other protein-coding genes are classified as intergenic lncRNAs; (ii) lncRNAs totally in the some protein-coding loci are classified as intragenic lncRNAs; and (iii) antisense lncRNAs overlapping with exons of a protein-coding transcript on the opposite strand.

### Differential expression of lncRNAs between MT and WT

The expression levels of the assembled transcripts were calculated and normalized using fragments per kilobase of transcript per million fragments (RPKM) using Cufflinks v2.1.1^49^. Transcripts with mapping coverage of less than half the transcript length and transcripts with FPKM < 1 were removed. The change in lncRNA expression was calculated as the fold change (FC): FC = FPKM of WT/FPKM of MT. Only the lncRNAs that met the criteria of |log_2_^FC^| ≥ 1 with *P* < 0.05 were considered differentially expressed lncRNAs. To identify the protein-coding gene expression patterns in the two genotypes, we mapped the reads against the citrus genome^32^ using bowtie2 software^57^. Expression levels for each gene were calculated by quantifying the reads according to the RPKM method^58^. Replicates were examined independently for statistical analysis. Genes that were differentially expressed by at least two-fold were tested for false discovery rate correlations at *P* ≤ 0.05^59^.

### Real-time PCR analysis

To validate the differentially expressed lncRNAs and genes, real-time PCR was performed. Primers for real-time PCR are listed in Table S7. Real-time PCR was performed based on a previously reported method^60^. Three biological replicates and four mechanical repetitions were assayed, and it was shown that they had similar trends. Data from one biological repeat are presented. The data were processed using one-way analysis of variance (ANOVA); significant differences were compared based on Student’s *t*-test. *P* < 0.01 was considered significant.

### Prediction of miRNA targets and miRNA endogenous target mimics from lncRNAs

Trifoliate orange lncRNAs were predicted as miRNA targets using psRobot^61^. The target mimics were predicted using psRobot combined with local scripts and the rules established by Wu^35^. Generally, the following rules were used: (1) bulges were only permitted at the 5′ end of the ninth to twelfth positions of the miRNA sequence; (2) the bulge in eTMs should be composed of only three nucleotides; (3) perfect nucleotide pairing was required at the 5′ end of the second to eighth positions of the miRNA sequence; and (4) except for the central bulge, the total mismatches and G/U pairs within eTM and miRNA pairing regions should be no more than three.

### Plasmid construction and genetic transformation

The genomic sequence containing the Pt-miR156a1/2-fold back region was amplified from genomic DNA as a template by using PCR. The PCR products were digested and cloned into *Sal* I and *BamH* I sites of vector pBI121 to generate the overexpression construct Pt-miR156a1 and Pt-miR156a2 (Table S7). The cloned vector was verified by sequencing. The targets (TCONS_00030665) from Pt-miR156 were cloned into vector pBI121 based on the same method. For *Arabidopsis* transformation, the floral dipping transformation method was used in this experiment^62^. T_3_ homozygous lines were used for all experiments presented. Identification and phenotype analysis of the transformants were performed using a previously reported method^46^, and the data were analyzed using Excel (Microsoft, Redmond, WA, USA).

## Additional Information

**How to cite this article:** Wang, C.-Y. *et al*. Genome-wide screening and characterization of long non-coding RNAs involved in flowering development of trifoliate orange (*Poncirus trifoliata* L. Raf.). *Sci. Rep.* **7**, 43226; doi: 10.1038/srep43226 (2017).

**Publisher's note:** Springer Nature remains neutral with regard to jurisdictional claims in published maps and institutional affiliations.

## Supplementary Material

Figure S1

Table S1

Table S2

Table S3

Table S4

Table S5

Table S6

Table S7
