# Supplementary material for: Genome-wide screening and characterization of long non-coding RNAs involved in flowering development of trifoliate orange (Poncirus trifoliata L. Raf.)
Source: Sci Rep. 2017 Feb 24;7:43226. doi: 10.1038/srep43226 (PMC5324131; doi:10.1038/srep43226)
Supplement: Figure S1 [file srep43226-s1.pdf]

Genome-wide screening and characterization of long non-coding RNAs involved in flowering development of trifoliate orange (*Poncirus trifoliata* L. Raf.)

Chen-Yang Wang[1], Sheng-Rui Liu[1,2], Xiao-Yu Zhang[1], Yu-Jiao Ma[1], Chun-Gen Hu[1,]*, Jin-Zhi Zhang[1,]*

[1]Key Laboratory of Horticultural Plant Biology (Ministry of Education), College of Horticulture and Forestry Science, Huazhong Agricultural University, Wuhan 430070, China

[2]Present address: State Key Laboratory of Tea Plant Biology and Utilization, Anhui Agricultural University, Hefei 230036, China

[1]These authors contributed equally to this work.

*Corresponding author

E-mail: jinzhizhang@mail.hzau.edu.cn or chungen@mail.hzau.edu.cn

Tel: +86-27-8728-1826

Fax: +86-27-8728-2010

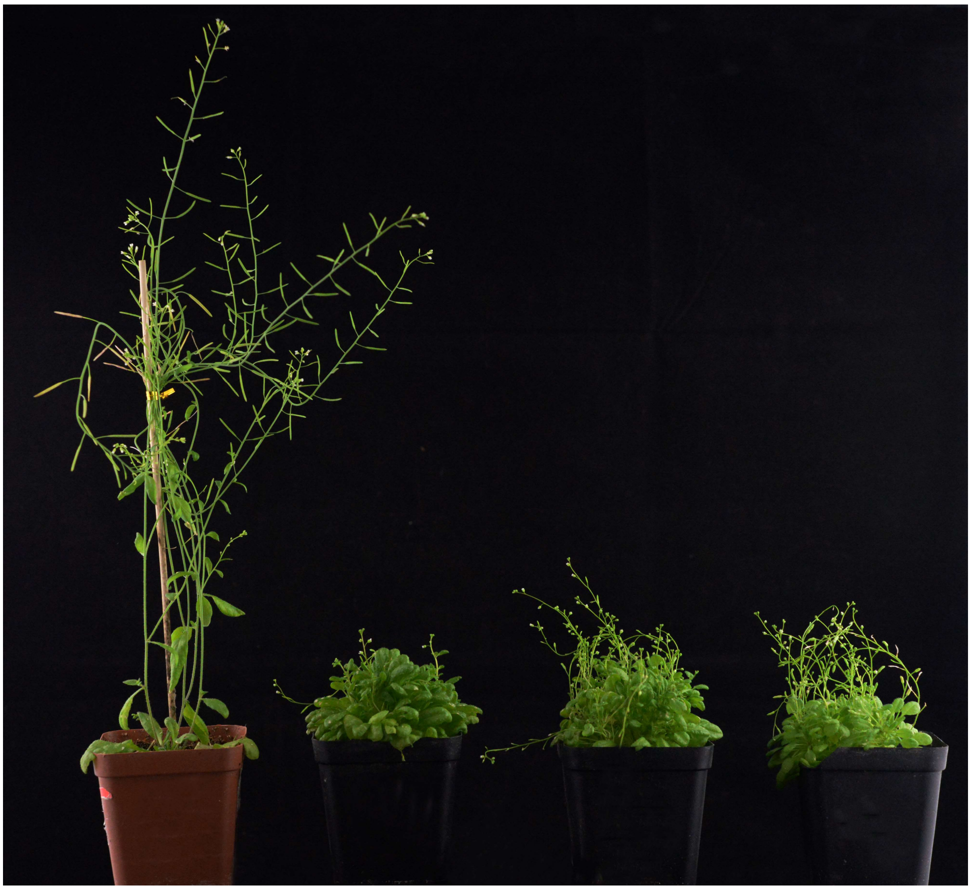

Figure S1. Senescent phenotypes of control and three transgenic lines of p35S:Pt-miR156a2.
